# Multimodality imaging methods and systemic biomarkers in classical low-flow low-gradient aortic stenosis: Key findings for risk stratification

**DOI:** 10.3389/fcvm.2023.1149613

**Published:** 2023-04-27

**Authors:** Maria Antonieta Albanez A. de M. Lopes, Carlos M. Campos, Vitor Emer Egypto Rosa, Roney O. Sampaio, Thamara C. Morais, Fábio Sândoli de Brito Júnior, Marcelo L. C. Vieira, Wilson Mathias, Joao Ricardo Cordeiro Fernandes, Antonio de Santis, Luciano de Moura Santos, Carlos E. Rochitte, Davide Capodanno, Corrado Tamburino, Alexandre Abizaid, Flavio Tarasoutchi

**Affiliations:** ^1^ Instituto do Coracao (InCor), Hospital das Clinicas HCFMUSP, Faculdade de Medicina, Universidade de Sao Paulo, Sao Paulo, SP, Brazil; ^2^ Real Hospital Português, Real Cardiologia, Recife, PE, Brazil; ^3^ Insituto Prevent Senior, São Paulo, SP, Brazil; ^4^ CardioT Division of Cardiology, Policlinico-Vittorio Emanuele Hospital University of Catania, Catania, Italy

**Keywords:** multimodality imaging, low-flow low-gradient aortic stenosis, B-type natriuretic peptide, high-sensitivity troponin I, biomarkers

## Abstract

**Objectives:**

The aim of the present study is to assess multimodality imaging findings according to systemic biomarkers, high-sensitivity troponin I (hsTnI) and B-type natriuretic peptide (BNP) levels, in low-flow, low-gradient aortic stenosis (LFLG-AS).

**Background:**

Elevated levels of BNP and hsTnI have been related with poor prognosis in patients with LFLG-AS.

**Methods:**

Prospective study with LFLG-AS patients that underwent hsTnI, BNP, coronary angiography, cardiac magnetic resonance (CMR) with T1 mapping, echocardiogram and dobutamine stress echocardiogram. Patients were divided into 3 groups according to BNP and hsTnI levels: Group 1 (*n* = 17) when BNP and hsTnI levels were below median [BNP < 1.98 fold upper reference limit (URL) and hsTnI < 1.8 fold URL]; Group 2 (*n* = 14) when BNP or hsTnI were higher than median; and Group 3 (*n* = 18) when both hsTnI and BNP were higher than median.

**Results:**

49 patients included in 3 groups. Clinical characteristics (including risk scores) were similar among groups. Group 3 patients had lower valvuloarterial impedance (*P* = 0.03) and lower left ventricular ejection fraction (*P* = 0.02) by echocardiogram. CMR identified a progressive increase of right and left ventricular chamber from Group 1 to Group 3, and worsening of left ventricular ejection fraction (EF) (40 [31–47] vs. 32 [29–41] vs. 26 [19–33]%; *p* < 0.01) and right ventricular EF (62 [53–69] vs. 51 [35–63] vs. 30 [24–46]%; *p* < 0.01). Besides, there was a marked increase in myocardial fibrosis assessed by extracellular volume fraction (ECV) (28.4 [24.8–30.7] vs. 28.2 [26.9–34.5] vs. 31.8 [28.9–35.5]%; *p* = 0.03) and indexed ECV (iECV) (28.7 [21.2–39.1] vs. 28.8 [25.4–39.9] vs. 44.2 [36.4–51.2] ml/m^2^, respectively; *p* < 0.01) from Group 1 to Group 3.

**Conclusions:**

Higher levels of BNP and hsTnI in LFLG-AS patients are associated with worse multi-modality evidence of cardiac remodeling and fibrosis.

## Introduction

Low-flow, low-gradient aortic stenosis (LFLG-AS) with reduced ejection fraction is the term used to identify patients with aortic valve area (AVA) ≤ 1.0 cm^2^ associated with low mean transaortic gradient (<40 mmHg) and reduced left ventricular ejection fraction (LVEF < 50%) (1). This entity is described in up to 10% of the aortic stenosis population (1, 2). It has been shown that classical LFLG-AS patients have poor outcomes with conservative management but are also considered being at high risk of events for both transcatheter and surgical aortic valve replacement (3).

Imaging methods have fundamental role in the diagnosis and management of valvular heart disease. Cardiac magnetic resonance (CMR) can provide detailed information about the myocardial disease. CMR assesses the impact of high afterload pressures on myocardial function and can quantify extracellular volume expansion (ECV) using T1 mapping (4).

Circulating biomarkers are commonly used in clinical decision making for diagnosing, risk stratification and management of various cardiovascular diseases (5). Several studies have showed strong relationships between the B-type natriuretic peptide (BNP) level and symptom development, left ventricular (LV) hypertrophy, LV function, severity of aortic stenosis and mortality (6). Although high-sensitivity troponin I (hsTnI) plasma levels are not mentioned in current guideline recommendations, combined measure of BNP and hsTnI have been recognized as predictors of adverse outcomes in LFLG-AS patients (7–9).

Although both biomarkers and imaging methods have shown prognostic implications, literature remains scarce on the relationship between them in LFLG-AS patients. Therefore, the aim of the present study was to assess the relationship between multimodality imaging methods and cardiac biomarkers (BNP and hsTnI) to help clarify the diagnosis and prognosis of patients with LFLG-AS.

## Methods

### Study population

**Figure 1:**
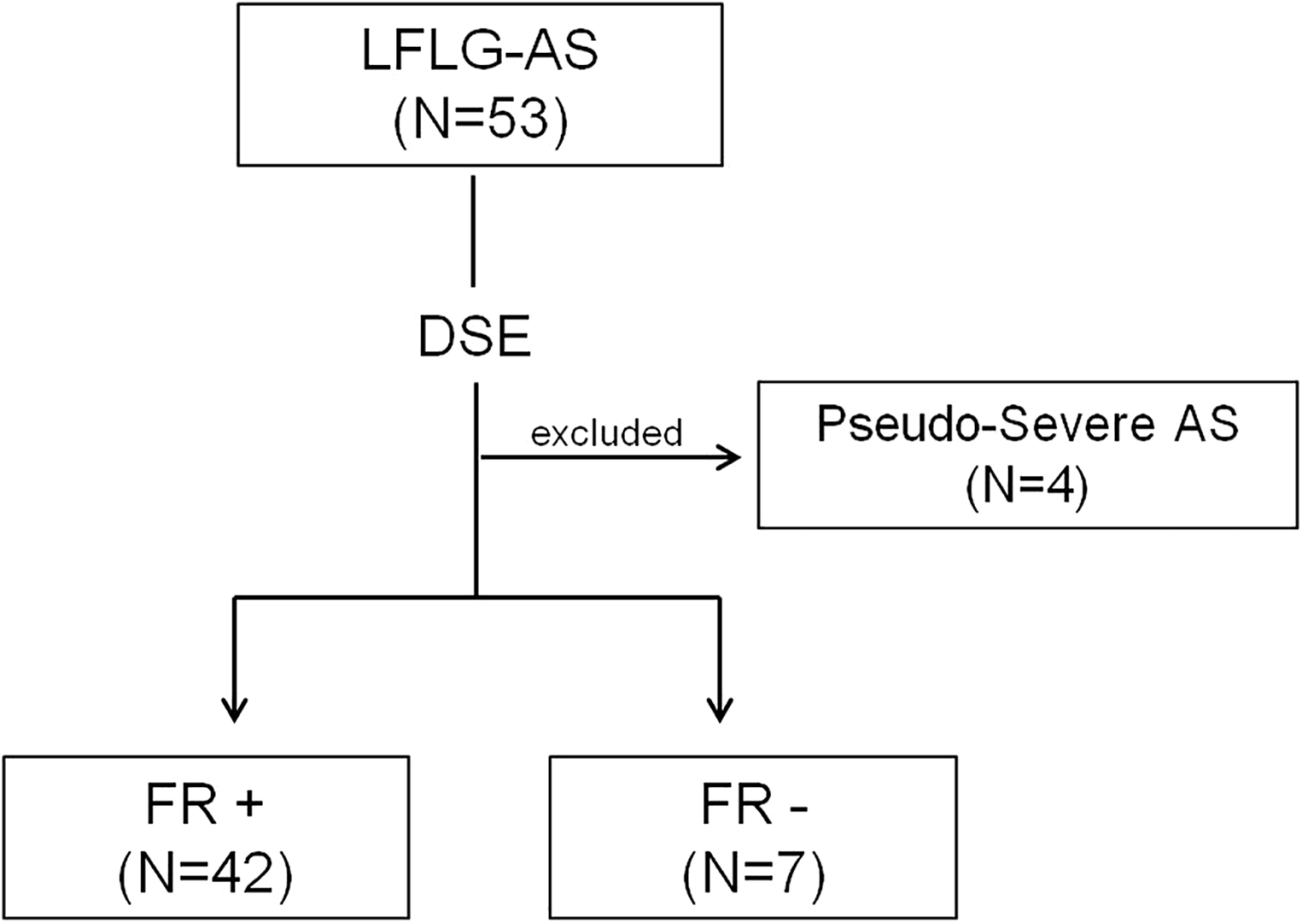
Study flowchart. Selection of the study population. AS, aortic stenosis; DSE, dobutamine stress echocardiography; FR, flow reserve; and LFLG, low-flow, low-gradient.

Patients with symptomatic LFLG-AS defined as mean gradient < 40 mmHg and indexed AVA ≤ 0.6 cm^2^/m^2^, with reduced LVEF (< 50%) were enrolled (*n* = 49; Figure 1). Exclusion criteria were: (I) previous valve surgery, (II) severe aortic regurgitation, (III) CMR incompatible devices or contraindications to gadolinium, (IV) severe primary mitral valve disease, (V) nonischemic cardiomyopathies, or (VI) diagnosis of pseudo-severe aortic stenosis on dobutamine stress echocardiogram. The study protocol was reviewed and approved by the local institutional ethics committee. All patients provided written informed consent.

### Study protocol

All of the patients with LFLG-AS underwent dobutamine stress echocardiogram, transthoracic echocardiography, T1 mapping and late gadolinium enhancement (LGE) CMR, and laboratory tests, including hsTnI (ADVIA Centaur TnI-Ultra; Siemens Healthcare Diagnostics, Tarrytown, NY; reference value: 0.015 ng/ml) and BNP (ADVIA Centaur; Siemens Medical Solutions Diagnostic, Los Angeles, CA, reference value: 200 pg/ml). Patients were divided in 3 groups according to BNP and hsTnI levels:

- Group 1 (low biomarkers group): Patients with both BNP *and* hsTnI below the median value (BNP < 395 pg/ml [< 1.98 folds upper reference value] *and* hsTnI < 0.042 ng/ml [<2.8 folds]);
- Group 2 (intermediate biomarkers group): Patients with either BNP *or* hsTnI higher or equal than the median value (BNP ≥ 395 pg/ml [≥1.98 folds upper reference value] or hsTnI ≥ 0.042 ng/ml [≥2.8 folds upper reference value]);
- Group 3 (high biomarkers group): Patients with *both* BNP and hsTnI higher or equal than the median value (BNP ≥ 395 pg/ml [≥1.98 folds upper reference value] and hsTnI ≥ 0.042 ng/ml [≥2.8 folds upper reference value]).

### Coronary angiography

All patients underwent coronary angiography, and coronary artery disease was defined as the presence of >50% luminal stenosis on major epicardial coronary arteries.

### Echocardiography

All transthoracic Doppler-echocardiographic exams were analyzed in a central echocardiography laboratory at our Institution. All echocardiographic parameters measured using the methods recommended by the American Society of Echocardiography (10, 11). Dobutamine stress echocardiogram was performed as previously described using a commercially available ultrasound system (Vivid 9; GE Healthcare, Milwaukee, WI) (12). Briefly, the dobutamine infusion protocol consisted of 5-min increments of 2.5 to 5 µg/kg per minute up to a maximum dosage of 20 µg/kg per minute. A minimum of 3 consecutive cycles were recorded. In patients with flow reserve (defined as the percentage increase in stroke volume index ≥20%), the presence of true-severe aortic stenosis was defined by a mean transaortic gradient ≥ 40 mmHg with an AVA ≤1.0 cm^2^ during dobutamine stress (12). In patients without flow reserve, aortic valve calcium score on computed tomography was used to confirm aortic stenosis severity (≥1,300 AU in women and ≥2000AU in men) (12–14). All flow reserve echocardiographic parameters were measured in a mean of 10 consecutive heart beats in patients with atrial fibrillation (11). Valvularterial impedance was calculated using the following formula: (systolic arterial pressure + mean transaortic gradient)/stroke volume index. Left ventricular global longitudinal strain was measured by speckle tracking with dedicated commercial software (EchoPAC V 110.0.x; GE Healthcare, Milwaukee, WI) as previously reported (15). Global longitudinal strain data were expressed in absolute value (|%|) and were defined as the mean of longitudinal strain of the 2-chamber, 3-chamber, and 4-chamber apical view.

### CMR protocol

**Figure 2:**
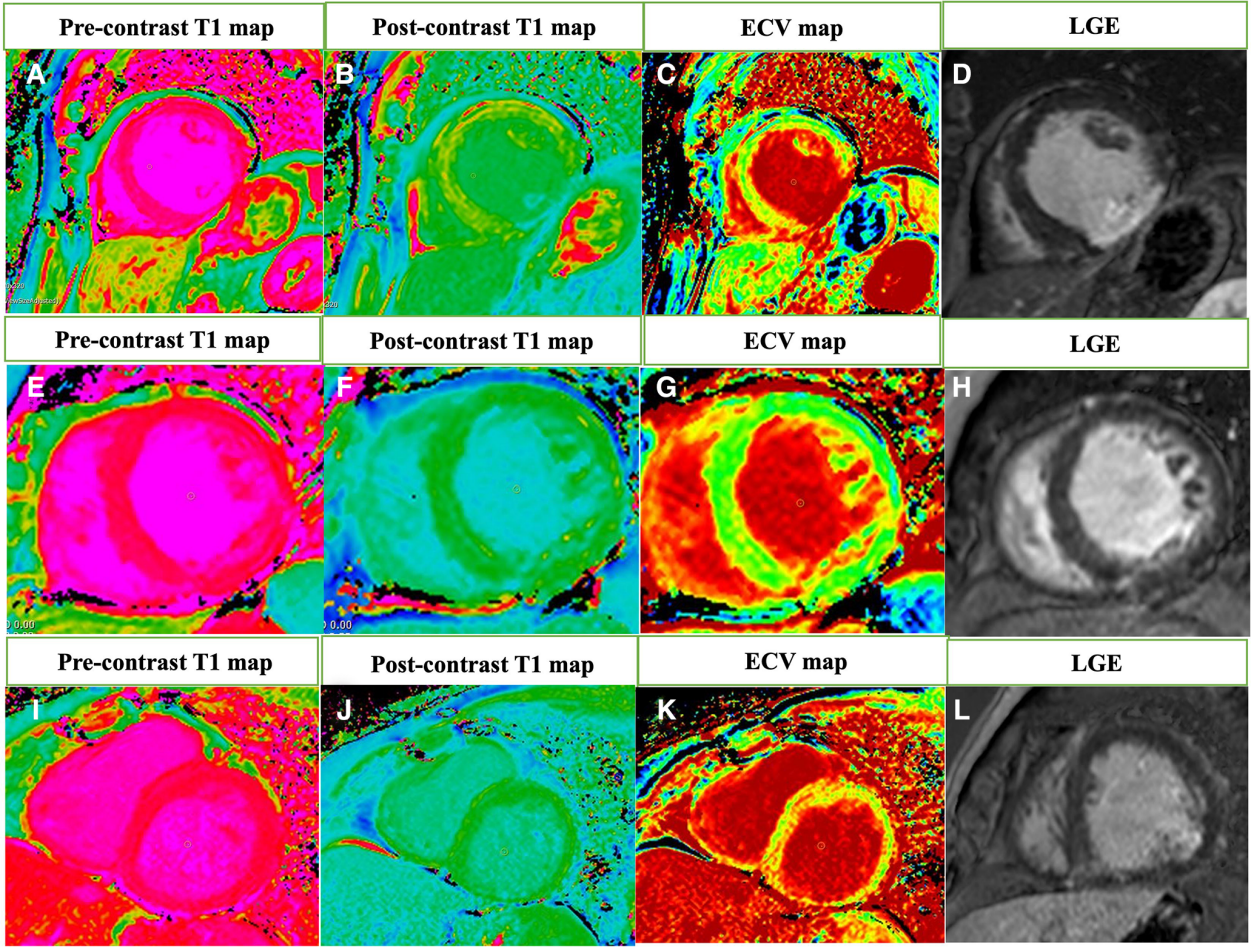
The extracellular volume (ECV) fraction calculation using the T1 mapping cardiovascular magnetic resonance and late gadolinium enhancement imaging. Native T1 map (**A**) and postcontrast T1 map (**B**) were used to calculate the ECV map (**C**) of a Group 1 patient with ECV = 8.9% and transmural late gadolinium enhancement imaging (LGE) in inferior and inferolateral walls (**D**—red arrow). Native T1 map (**E**) and postcontrast T1 map (**F**) of a Group 2 patient with ECV = 32.0% (**G**) and LGE in inferior ventricular insertion point (**H**—red arrow). Native T1 map (**I**) and postcontrast T1 map (**J**) of a Group 3 patient with ECV = 43.5% (**K**) and subendocardial LGE in inferolateral wall (**L**—red arrow).

All patients underwent CMR using a clinical 1.5-T CMR scanner (Achieva; Philips, Best, the Netherlands), and the analyses were performed by 2 investigators in a central CMR core laboratory at our Institution, blinded to clinical and echocardiographic parameters, as previously described (12). LGE images were acquired using a phase-sensitive inversion recovery sequence and the inversion time was individually determined to null the normal myocardial signal. LGE quantification was obtained with thresholding technique by 3 standard deviations above remote myocardium. T1 mapping MOLLI images were acquired pre and 15 min after gadolinium injection in 3 short-axis images (basal, mid-ventricular, and apical levels). T1 mapping analysis were performed including and excluding areas of LGE. Endocardial and epicardial delineations, including LGE areas, were manually traced in all 3 short-axis images, for global myocardial T1 calculation (pre- and post-gadolinium images). Subendocardial and transmural LGE areas were manually excluded using a region of interest (ROI) delimitation tool, for non-enhanced myocardium T1 calculation (pre- and post-gadolinium images). An additional ROI was placed at the center of the LV cavity, for blood pool T1 calculation. In patients with atrial fibrillation, T1 mapping image acquisition was repeated, and an average of T1 values was calculated in both pre- and post-gadolinium sequences. Besides, all these patients had controlled heart rate (60–90 bpm) at the time of CMR (16).The extracellular volume fraction (ECV) (for global and for non-enhanced myocardium) was calculated using a correction for blood hematocrit (collected on the same day of CMR acquisition), as follows: ECV = (1−hematocrit) * (ΔR1 myocardium/ΔR1blood pool). Where ΔR1 = (1/T1post-gadolinium − 1/T1pre-gadolinium). We also calculated the indexed ECV (iECV) of non-enhanced myocardium using the following formula: ECV × indexed LV end-diastolic myocardial volume, as previously described (4) (Figure 2).

### Statistical analysis

Continuous variables were presented as mean ± standard deviation or median (interquartile range) and Shapiro-Wilk test was used to test the normality of variables. Categorical variables were presented as percentages. Log transformation was applied to normalize data distribution. Kruskall-Wallis, or ANOVA test was applied for continuous variables, and chi-square test and Fisher exact test was applied for categorical variables, as appropriate. The *post hoc* analysis was performed with Tukey test. Spearman correlation coefficients were used to evaluate data correlation. ROC curve was applied for BNP and hsTnI to discriminate increase in ECV, iECV, left ventricle end-systolic volume index (LVESVi), left ventricle end-diastolic volume index (LVEDVi), right ventricle end-systolic volume index (RVESVi), right ventricle end-diastolic volume index (RVEDVi), right ventricle ejection fraction (RVEF), and left ventricle ejection fraction (LVEF), using cutoff values established in the literature (ECV > 28% (17); iECV > 22.5 ml/m^2^; LVESVi ≥ 29 ml/m^2^ for women and ≥36 ml/m^2^ for men; LVEDVi ≥ 74 ml/m^2^ for women and ≥85 ml/m^2^ for men; RVESVi ≥ 33 ml/m^2^ for women and ≥43 ml/m^2^ for men; RVEDVi ≥ 77 ml/m^2^ for women and ≥93 ml/m^2^ for men; RVEF < 55%; and LVEF < 55% (4, 18). All tests were 2 tailed, and a P < 0.05 was used to indicate statistical significance. All analyses were conducted using the statistical package SPSS, version 23 (IBM, Armonk, NY).

## Results

### Baseline patients data

The main clinical and laboratory data are shown in Table 1. Among the 49 patients included in the present study, the mean age was 67.4 ± 8.4 years, 38 patients (77%) were male, 34 (69%) had hypertension, 18 (37%) had diabetes, and 18 (37%) had coronary artery disease. The mean STS score was 3.24 ± 2.08% and the mean EuroSCORE II was 3.39 ± 2.66%.

**Table 1:** Baseline clinical and laboratory data of the study population.

	Group 1 Low biomarkers (*n* = 17)	Group 2 Intermediate biomarkers (*n* = 14)	Group 3 High Biomarkers (*n* = 18)	*P* value*
Age, years	66.65 ± 6.6	71.07 ± 9.6	65.44 ± 8.5	0.16
Body surface area, m^2^	1.82 ± 0.16	1.77 ± 0.12	1.82 ± 0.17	0.42
Female gender	6 (35.3)	3 (21.4)	2 (11.1)	0.22
Diabetes Mellitus	7 (41.2)	6 (42.9)	5 (27.8)	0.61
Hypertension	14 (82.4)	10 (71.4)	10 (55.6)	0.22
Atrial fibrillation	4 (23.5)	3 (21.4)	5 (27.8)	0.91
NYHA III/IV	7 (14.3)	9 (18.4)	12 (24.5)	0.26
Angina	2 (11.8)	6 (42.9)	4 (22.2)	0.36
Coronary artery disease	6 (35.3)	5 (35.7)	7 (38.9)	0.97
One vessel	2 (11.8)	2 (14.3)	0 (0)	0.39
Two vessels	1 (5.9)	2 (14.3)	2 (11.1)	0.39
Three vessels	3 (17.6)	1 (7.1)	5 (37.8)	0.39
Previous CABG	3 (17.6)	1 (7.1)	3 (16.7)	0.66
EuroSCORE II, %	2.82 ± 2.5	3.36 ± 1.8	4.03 ± 3.3	0.31
STS, %	2.87 ± 2.1	3.76 ± 2.1	3.16 ± 1.9	0.26
**Medications**				
ACE inhibitor or ARB	16 (94.1)	10 (71.4)	9 (50)	<0.01^‡^
Beta blockers	12 (70.6)	4 (28.6)	10 (55.6)	0.06
Antiplatelets	13 (76.5)	10 (71.4)	8 (44.4)	0.11
Diuretics	14 (82.4)	12 (85.7)	17 (94.4)	0.50
Statins	16 (94.1)	9 (64.3)	11 (61.1)	0.03
Digital	2 (11.8)	3 (21.4)	4 (22.2)	0.68
Oral anticoagulation	4 (23.5)	2 (14.3)	6 (33.3)	0.45
**Electrocardiogram**				
Left Bundle Branch Block	5 (29.4)	5 (35.7)	3 (16.7)	0.44
Right Bundle Branch Block	0 (0)	2 (14.3)	1 (5.6)	0.19
**Laboratory data**				
Hematocrit, %	39.4 ± 10.3	40.6 ± 5.0	41.6 ± 6.6	0.70
C reactive protein, pg/ml	3.16 (1.14–3.12)	9.54 (2.43–11.47)	13.04 (1.41–14.02)	0.12
eGFR, ml/min	67.7 ± 22.7	47.0 ± 17.0	50.7 ± 22.5	0.01^†^
CKD (eGFR <60 ml/min)	3 (17.6)	6 (42.9)	9 (50)	0.12
High-sensitivity troponin I, ng/ml	0.01 (0.0085–0.02)	0.12 (0.03–0.17)	0.16 (0.05–0.16)	<0.01^†^^‡^
B-type natriuretic peptide, pg/ml	148.87 (66–200)	498.35 (109.25–679.5)	1,245.94 (583.75–1,608)	<0.01^‡^^§^

Values are mean ± standard deviation, median (interquartile range), or n (%). ACE indicates angiotensin-converting enzyme; ARB, angiotensin receptor blocker; CABG, coronary artery bypass graft; CKD, chronic kidney disease; eGFR, estimated glomerular filtration rate.

* Overall *P* value among groups: group 1, group 2 and group 3.

^†^ Significant difference (*P *< 0.05) between group 1 vs. group 2.

^‡^ Significant difference (*P *< 0.05) between group 1 vs. group 3.

^§^ Significant difference (*P *< 0.05) between group 2 vs. group 3.

**Figure 3:**
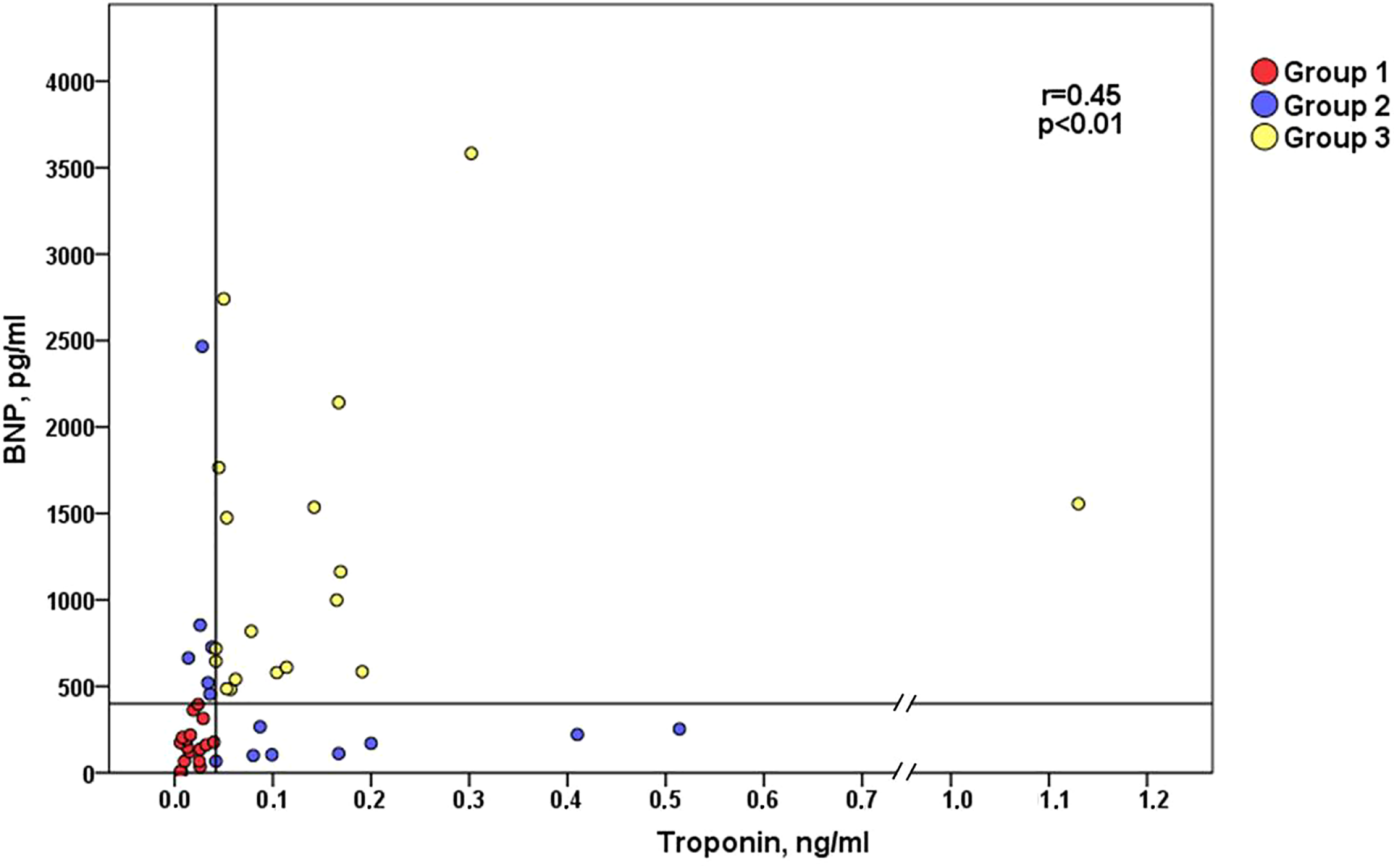
Correlation between B-type natriuretic peptide (BNP) and troponin I (hs-TnI). Groups were divided according to biomarkers median levels: Group1 patients (red dots) with BNP and hs-TnI below median; Group 2 patients (blue dots) with BNP or hs-TnI above median; and Group 3 patients (yellow dots) with both BNP and hs-TnI above median. *r = Spearman correlation coefficient.

Seventeen patients were in the low biomarkers group (Group 1), 14 in the intermediate (Group 2) and 18 in the high biomarkers group (Group 3). Overall, the baseline clinical characteristics among groups are in Table 1. The baseline characteristics are similar among 3 groups. One exception is the use of angiotensin-converting enzyme inhibitors/angiotensin receptor blocker and statin. Also, estimated glomerular filtration rate was also different between groups (67.7 ± 22.7 vs. 47.0 ± 17.0 vs. 50.7 ± 22.5 ml/min, respectively; *p* = 0.01) with significant difference between groups 1 and 2 (*p* = 0.02 for *post hoc* test). As shown in Figure 3, hsTnI and BNP had positive correlation (*r* = 0.450, *p* < 0.01). Baseline characteristics according isolated BNP and hsTnI tertiles are shown in Supplementary Tables S1, S2, respectively.

### Baseline echocardiography and echocardiographic stress data

Table 2 depicts echocardiographic findings at baseline. Overall, the median LVEF was 34% [28–41], the median AVA was 0.88 [0.70–0.96] cm^2^, and the mean transaortic gradient was 25 ± 7 mmHg. At baseline echocardiogram, we found LVEF (37.0 [32.5–43.5] vs. 35.5 [28.0–40.8] vs. 28.0 [22.0–37.5]%, respectively; *p* = 0.02), lower in group 3 compared to group 1 (*p* = 0.01 for *post hoc* analysis), and valvuloarterial impedance (5.2 [4.8–5.5] vs. 5.7 [5.0–6.6] vs. 4.8 [4.4–5.3] mmHg/ml/m^2^, respectively; *p* = 0.03), with significant difference between groups 2 and 3 (*p* = 0.02 for *post hoc* analysis). Interestingly, there were no significant difference regarding the presence of flow reserve between the groups (76.5% vs. 100.0% vs. 88.2%, respectively; *p* = 0.07). Baseline echocardiography and echocardiographic stress data according isolated BNP and hsTnI tertiles are shown in Supplementary Tables S3, S4, respectively. Among patients with no FR, the severity of AS was assessed by computed tomography in all patients, and the median valve calcium score was 1,885 (1,291–5,875) AU.

**Table 2:** Baseline echocardiography and dobutamine stress echocardiography data.

	Group 1 Low biomarkers (*n* = 17)	Group 2 Intermediate biomarkers (*n* = 14)	Group 3 High biomarkers (*n* = 18)	*P* value*
**Baseline Echocardiography**				
Aortic root, mm	33.0 (29.0–37.0)	31.5 (30.0–34.25)	32.0 (30.0–35.5)	0.92
Left atrium, mm	45.0 ± 5.9	46.0 ± 5.5	50.0 ± 6.8	0.06
Interventricular septum, mm	11.0 ± 1.9	11.2 ± 2.9	11.1 ± 1.7	0.98
Posterior wall, mm	10.0 (9.0–11.0)	11.0 (9.75–12.0)	10.0 (9.5–11.0)	0.55
LVEDV, mm	56.82 ± 5.60	58.42 ± 7.10	60.66 ± 7.60	0.26
LVESV, mm	44.82 ± 6.50	45.71 ± 6.50	49.88 ± 8.90	0.13
LVEF, %	37.0 (32.5–43.5)	35.5 (28.0–40.8)	28.0 (22.0–37.5)	0.02^†^
LV mass, g	136.90 ± 26.30	148.78± 28.40	167.72± 65.90	0.14
Stroke volume index, ml/m^2^	33.5 (31.0–44.8)	31.5 (27.8–39.3)	37.0 (29.0–42.9)	0.50
Aortic valve area, cm^2^	0.87 (0.80–1.00)	0.81 (0.65–0.95)	0.82 (0.70–0.95)	0.37
Aortic valve area index, cm^2^/m	0.48 ± 0.10	0.45 ± 0.00	0.44 ± 0.00	0.51
Peak transaortic gradient, mmHg	41.76 ± 13.60	45.92 ± 12.30	42.66 ± 13.10	0.66
Mean transaortic gradient, mmHg	24.29 ± 8.90	27.14 ± 6.50	25.00 ± 8.00	0.60
Moderate/severe functional mitral regurgitation	6.0 (35.3)	5.0 (35.7)	6.0 (33.3)	0.98
Moderate/severe functional tricuspid regurgitation,	3.0 (17.6)	2.0 (14.3)	2.0 (11.1)	0.85
Systolic pulmonary artery pressure, mmHg	41.4 ± 12.0	46.0 ± 11.0	43.8 ± 9.9	0.70
Valvuloarterial impedance, mmHg/ml/m^2^	5.2 (4.8–5.5)	5.7 (5.0–6.6)	4.8 (4.4–5.3)	0.03^‡^
Global longitudinal strain, [−]%	10.29 ± 2.40	10.43 ± 3.20	8.98 ± 2.20	0.25
**Dobutamine Stress Echocardiography**				
Basal aortic valve area, cm^2^	0.90 (0.78–1.05)	0.80 (0.75–0.89)	0.90 (0.60–1.00)	0.21
Peak stress aortic valve area, cm^2^	0.98 (0.79–1.40)	0.86 (0.80–1.00)	0.84 (0.67–1.00)	0.89
Basal mean transaortic gradient, mmHg	25.69 ± 8.10	28.31 ± 8.60	26.00 ± 8.90	0.68
Peak stress mean gradient, mmHg	37.37 ± 11.50	38.77 ± 9.70	44.00 ± 21.60	0.63
Basal stroke volume index, ml/m^2^	34.84 ± 6.60	30.67 ± 9.40	35.02 ± 21.40	0.77
Peak stress stroke volume index, ml/m^2^	41.47 ± 7.10	40.36 ± 15.10	36.63 ± 7.80	0.56
Presence of flow reserve	13 (76.5)	14 (100.0)	15 (88.2)	0.07

Values are mean ± standard deviation, median (interquartile range), or n (%). LV means left ventricular; LVEDV, left ventricular end-diastolic volume; LVEF, left ventricular ejection fraction; and LVESV, left ventricular end-systolic volume.

* Overall *P* value among groups: group 1, group 2 and group 3.

^†^ Significant difference (*P* < 0.05) between group 1 vs. group 3.

^‡^ Significant difference (*P* < 0.05) between group 2 vs. group 3.

### Cardiac magnetic resonance imaging

**Figure 4:**
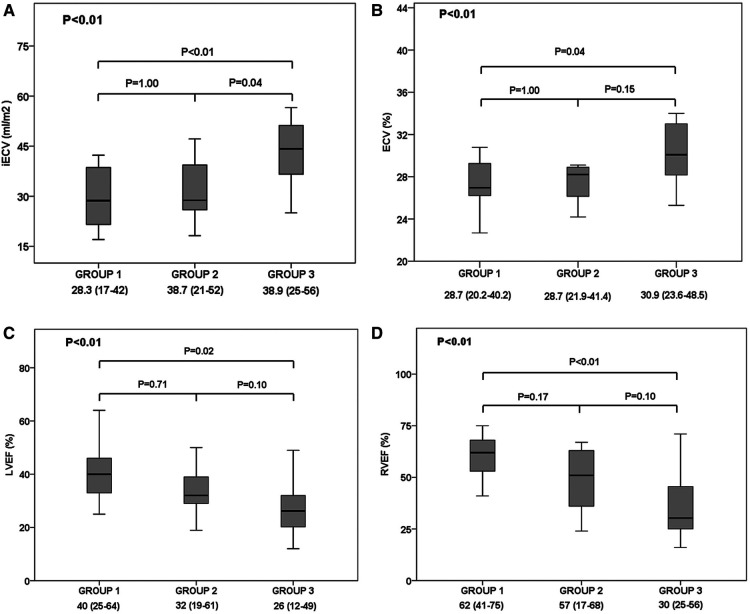
Cardiac magnetic resonance imaging's findings according to troponin I and B-type natriuretic peptide groups: Group 1 (low biomarkers group); Group 2 (intermediate biomarkers group; Group 3 (high biomarkers group). Comparison of (**A**) indexed extracellular volume fraction (iECV), (**B**) extracellular volume fraction (ECV), (**C**) left ventricular ejection fraction (LVEF), and (**D**) right ventricular ejection fraction (RVEF) between patients of group 1, 2 and 3. Solid horizontal line indicates mean value; gray box, 1SD; and vertical line, high and lowest mean values.

**Figure 5:**
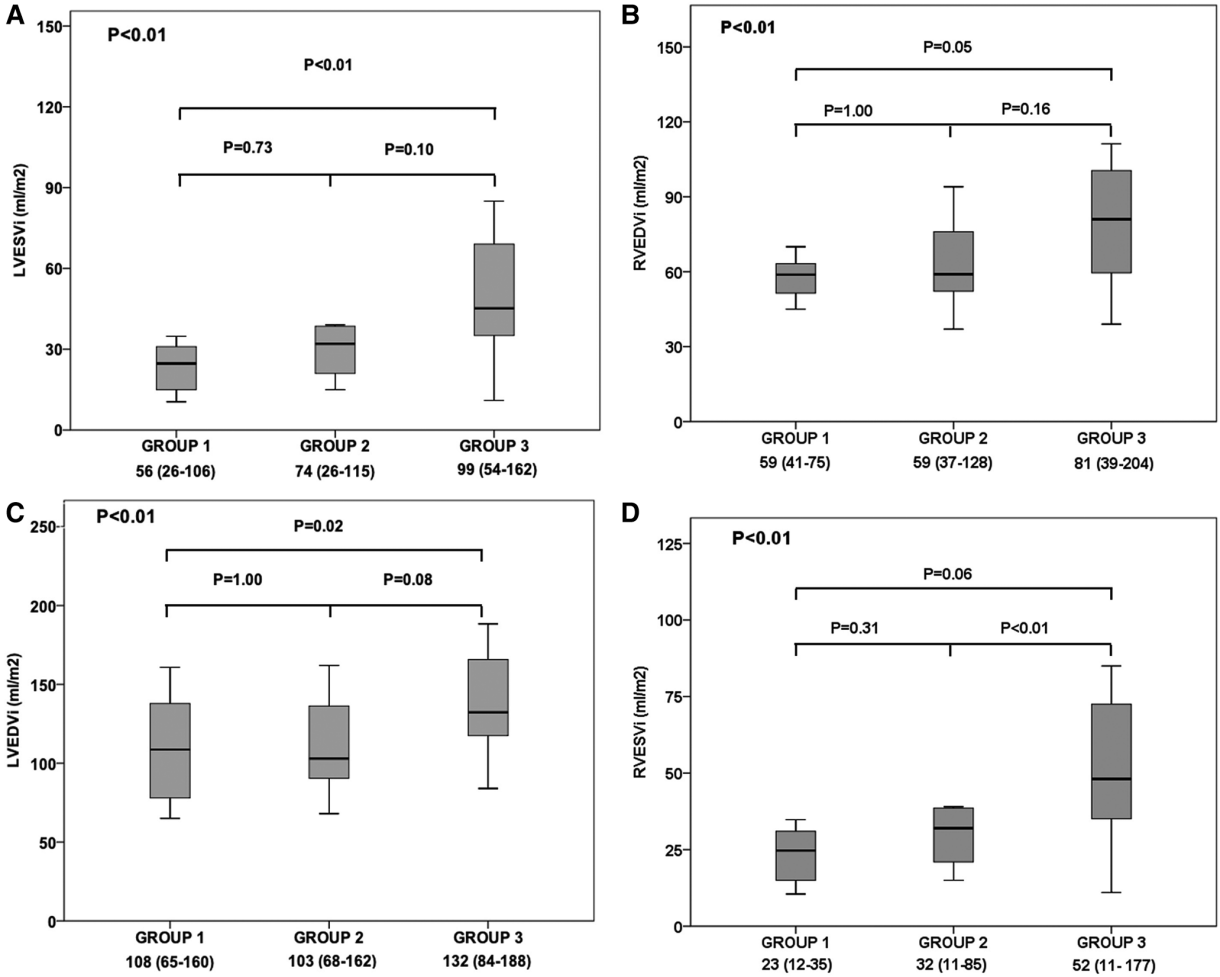
Cardiac magnetic resonance imaging's findings according to troponin I and B-type natriuretic peptide groups: Group 1 (low biomarkers group); Group 2 (intermediate biomarkers group; Group 3 (high biomarkers group). Comparison of (**A**) indexed left ventricular end-systolic volume (LVESVi), (**B**) indexed right ventricular end-diastolic volume (RVEDVi), (**C**) indexed left ventricular end-diastolic volume (LVEDVi), and (**D**) indexed right ventricular end-systolic volume (LVEDVi), between patients of group 1, 2 and 3. Solid horizontal line indicates mean value; gray box, 1SD; and vertical line, high and lowest mean values.

The CMR at baseline demonstrated various differences according to biomarkers levels groups (Table 3, Figures 4, 5). There was an increase in right and LV (systolic and diastolic) volumes from Group 1 to Group 3 (Figures 5A–D). Consequently, there was a deterioration of ejection fraction of both right and LV as biomarkers pattern worsened (Figures 4C,D). There were also differences in the groups regarding iECV (28.7 [21.2–39.1] vs. 28.8 [25.4–39.9] vs. 44.2 [36.4–51.2] ml/m^2^, respectively; overall *p* < 0.01; with *post hoc p* < 0.01 between groups 1 and 3 and *post hoc p* = 0.04 between groups 2 and 3) (Figure 4A) and ECV including delayed-enhancement images (28.4 [24.8–30.7] vs. 28.2 [26.9–34.5] vs. 31.8 [28.9–35.5]%, respectively; overall *p* = 0.03; with *post hoc p* = 0.02 between groups 1 and 3) (Figure 4B). Baseline CMR imaging data according isolated BNP and hsTnI tertiles are shown in Supplementary Tables S5, S6, respectively.

**Table 3:** Baseline cardiac magnetic resonance data.

	Group 1 Low biomarkers (*n* = 17)	Group 2 Intermediate biomarkers (*n* = 14)	Group 3 High biomarkers (*n* = 18)	*P* value*
RVEDV index, ml/m^2^	58.8 (48.2–65.6)	59.0 (50.6–82.5)	81.0 (59.3–100.8)	0.03^†^
RVESV index, ml/m^2^	24.7 (14.5–31.0)	32.0 (20.5–38.8)	48.1 (34.6–73.8)	<0.01^†^
RV ejection fraction, %	62.0 (53.0–69.0)	51.0 (35.2–63.0)	30.3 (24.0–46.3)	<0.01^†^
LVEDV index, ml/m^2^	108.7 (77.0–139.4)	103.0 (87.0–136.7)	132.3 (116.3- 167.1)	0.01^†^^‡^
LVESV index, ml/m^2^	65.0 (43.0–80.7)	74.0 (57.1–93.5)	99.0 (78.3–131.0)	0.01^†^
LVEF, %	40.0 (31.5–47.0)	32.0 (29.0–41.5)	26.2 (19.1–33.0)	<0.01^†^
Aortic valve area, cm^2^	0.84 ± 0.23	0.79 ± 0.22	0.89 ± 0.40	0.68
Positive transmural delayed-enhancement images	6.00 (35.30)	1.00 (7.70)	9.00 (56.30)	0.02^‡^
Positive mesocardial delayed-enhancement images	1.00 (5.90)	5.00 (38.50)	5.00 (31.30)	0.06
LV mass, g	187.70 ± 52.43	201.46 ± 41.58	211.55 ± 60.76	0.44
Late gadolinium enhancement mass, g	10.05 ± 13.04	8.30 ± 11.45	11.81 ± 11.91	0.52
ECV including delayed-enhancement images, %	28.4 (24.8–30.7)	28.2 (26.9–34.5)	31.8 (28.9–35.5)	0.03^†^
ECV without delayed-enhancement images, %	26.8 (26.2–29.3)	28.7 (26.2–32)	30.4 (28.2–33.7)	0.06
iECV, ml/m^2^	28.7 (21.2–39.1)	28.8 (25.4–39.9)	44.2 (36.4–51.2)	<0.01^†^^‡^

Values are mean ± standard deviation, median (interquartile range), or *n* (%). ECV indicates extracellular volume; iECV, indexed extracellular volume; LGE, late gadolinium enhancement; LV, left ventricular; LVEDV, left ventricular end-diastolic volume; LVEF, left ventricular ejection fraction; LVESV, left ventricular end-systolic volume; RV, right ventricular; RVEDV, right ventricular end-diastolic volume; and RVESV, right ventricular end-systolic volume.

* Overall *P* value among groups: group 1, group 2 and group 3.

^†^ Significant difference (*P* < 0.05) between group 1 vs. group 3.

^‡^ Significant difference (*P* < 0.05) between group 2 vs. group 3.

### Accuracy of BNP and hsTnI in the assessment of cardiac repercussion

**Figure 6:**
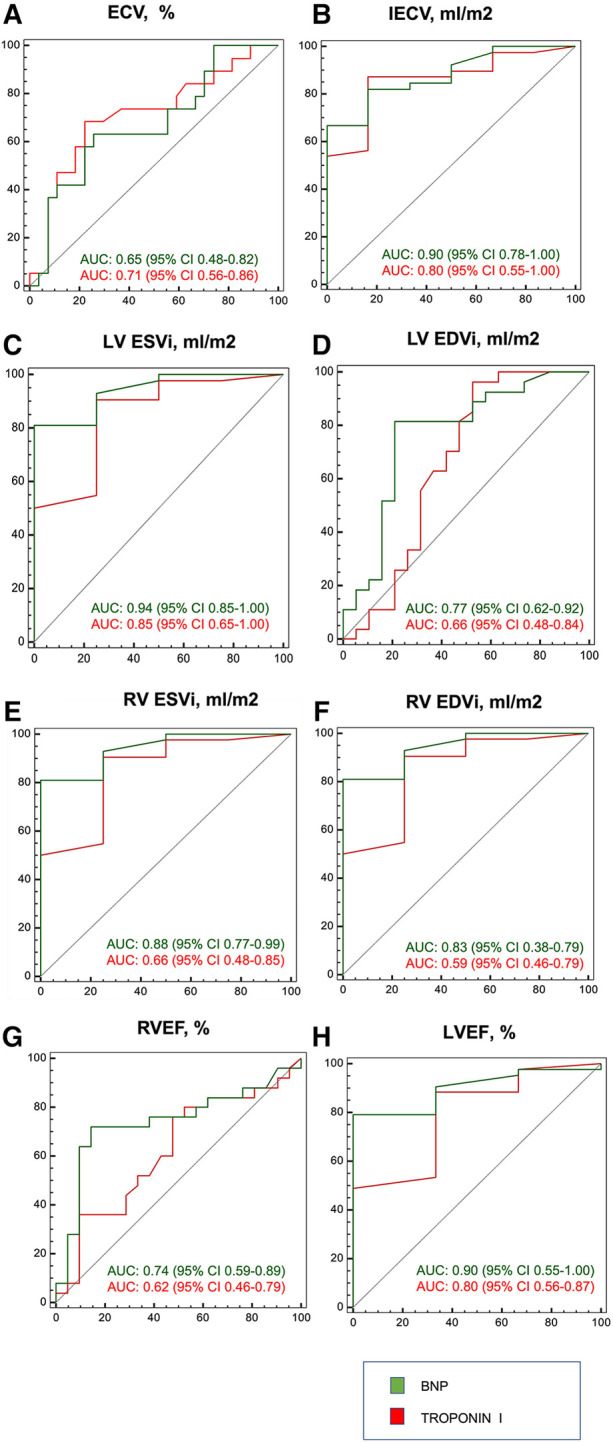
ROC curve of BNP and troponin I for cardiac magnetic resonance imaging findings. Biomarkers were tested for: (**A**) ECV > 28%; (**B**) iECV > 22.5 ml/m^2^; (**C**) LVESVi≥29 ml/m^2^ for women and ≥36 ml/m^2^ for men; (**D**) LVEDVi ≥ 74 ml/m^2^ for women and ≥85 ml/m^2^ for men; (**E**) RVESVi ≥ 33 ml/m^2^ for women and ≥43 ml/m^2^ for men; (**F**) RVEDVi ≥ 77 ml/m^2^ for women and ≥93 ml/m^2^ for men; (**G**) RVEF < 55%; and (**H**) LVEF < 55% (4, 18).

Both isolated BNP and hsTnI demonstrated excellent accuracy capacity to detect increase in iECV, LVESVi and LVEF (Figures 6B,C,H, respectively), and moderate capacity to detect increase in ECV, LVEDVi and RVEF (Figures 6A,D,G, respectively). BNP also had excellent discriminative capacity to detect increase in RVESVi and RVEDVi, however, hsTnI had only moderate capacity (Figures 6E,F, respectively).

## Discussion

The main findings of the present study can be summarized as follows: (1) In LFLG-AS patients, group classification according to BNP and hsTnI levels was associated with progressive worsening of imaging parameters of bi-ventricular remodeling and LV fibrosis by CMR; (2) the elevation of BNP and hsTnI was also associated with worse echocardiographic LVEF despite reduction on valvuloarterial impedance; (3) both BNP and hsTnI demonstrated good discriminative capacity to detect increase in LV parameters of function and fibrosis; (4) higher levels of BNP and hsTnI were not associated with flow reserve parameters.

Patients with LFLG-AS correspond to 5%–10% of patients with AS (1). They also represent a more challenging subgroup of AS patients both from a diagnostic and prognostic point of view. Previous series have shown high surgical mortality with an even worse prognosis with conservative therapy (19–22). Transcatheter aortic valve replacement (TAVR) is an attractive alternative due to its less invasive profile. It is associated with good short-term outcomes in LFLG-AS patients, however with a high 2-year mortality (23). There is a lack of prospective studies comparing surgical and TAVR in LFLG-AS, hindering prognostic stratification and risk assessment.

CMR is a novel tool in the prognostic assessment of AS patients. Focal fibrosis demonstrated by LGE has been shown to be a powerful mortality predictor (4, 24, 25). Besides, ECV and iECV are validated parameters to estimate diffuse histological fibrosis, and their association with LGE can also predict development of LV decompensation (4, 26). Compared to high-gradient AS, LFLG-AS have significantly higher ECV, iECV and LGE mass (12). Furthermore, CMR predicts mortality in LFLG-AS patients according to the number of impaired LV components as follows: presence of LGE, worsening of LV global longitudinal strain (>−11%), and increased ECV (>28%) (17). The greater the number of impaired components, the worse the outcomes, demonstrating the importance of LV functional and structural assessment in patients with LFLG-AS (17).

In this context, isolated troponin and BNP proved to be LV remodeling markers and mortality predictors, both in high-gradient AS and LFLG-AS (27–30). This predictive ability is an important issue, as assessment with biomarkers is easier and less expensive than by CMR. Thus, studies comparing both methodologies are increasingly necessary. However, data on combined LFLG-AS assessment of troponin, a marker of myocyte cell death, and BNP, a hormone released because of increased intracavitary cardiac pressure, are lacking. Dahou et al., in a recent study, evaluated the prognostic impact of combined troponin and BNP in 65 LFLG-AS patients and 33 paradoxical AS patients. It was demonstrated that the elevation of biomarkers had a prognostic impact. Furthermore, BNP alone was correlated with parameters of LV function and troponin correlated with parameters of LV geometry and function. Besides, this was an echocardiographic study that used predefined parameters of normality of biomarkers (BNP ≥ 550 pg/ml and high-sensitivity cardiac troponin T ≥ 15 ng/l) (30).

The present study is the first to evaluate the classification according to BNP and hsTnI levels as surrogated markers of progressive worsening of imaging parameters of bi-ventricular remodeling and LV fibrosis by CMR and echocardiography in a LFLG-AS cohort. As troponin assays may differ between centers, we chose to use folds of increase to define raises in these biomarkers (BNP above ∼2 folds and hsTnI above∼3 folds of reference values). Interestingly, Group 3 patients (increased BNP and hsTnI) had lower valvuloarterial impedance values and this finding can be explained by the reduction in ventricular function with a consequent tendency to reduce the mean transaortic gradient compared to the other groups.

As CMR is the gold standard for cardiac cavity and function measurements, the proposed group definition demonstrated that the progression of the groups was associated with worsening of parameters of cavity measurement and function by CMR, including interstitial fibrosis (31). ECV and iECV progression according to the groups reaffirms that the use of biomarkers correlates with marked cardiac prognostic changes, and the group definition can be used as a surrogate of structural heart disease. Indeed, isolated BNP and hsTnI had adequate capacity to detect increase in ECV and iECV, and reduced LVEF and RVEF. Therefore, as expected, the association of both elevated biomarkers defines a group with increased cardiac chambers and diffuse fibrosis, and possibly worse prognosis. Besides, T1 mapping CMR has an important role in the evaluation of differential diagnoses of cardiomyopathy, as cardiac amyloidosis. Thus, the patients included in the present study had a median ECV value of ≈30%, different of the 46.9% cutoff value used to rule out cardiac amyloidosis in Martinez-Naharro et al. study (32). These results confirm that, in our cohort, cardiac amyloidosis did not justify LFLG-AS phenotype. Also, despite similar echocardiographic pulmonary artery systolic pressure between the groups, such findings reinforce the hypothesis that RV dysfunction was a consequence of the AS excessive afterload mismatch, and not as a result of another cardiac disease.

Other important fact to mention is about flow reserve. LFLG-AS traditionally has been further divided according to the presence or absence of flow reserve on dobutamine stress echocardiography. Earlier series described poor prognosis in patients without flow reserve, suggesting that the absence of flow reserve was related to a more damaged LV (19–22). However, recent studies contradict these findings, demonstrating that such patients without flow reserve do not have more diffuse fibrosis compared to those with flow reserve, in addition to showing similar recovery of LVEF after valve intervention (12, 33). In agreement with such studies, there was no difference according to the group definition regarding the absence of flow reserve. Flow reserve characterization increasingly appears to be related only with AS severity confirmation and not for prognostic information.

### Limitations

This is an observational single-center study, with a relatively small number of patients, albeit large for this clinical entity. The effect of interventions in these patients, serial changes in biomarkers and imaging could not be tested. Besides, cutoff values were defined according to the median biomarkers values found in our population. However, despite small sample size, there were no differences in the groups regarding comorbidities and coronary artery disease, and this classification demonstrated good discriminative capacity to detect increase in LV parameters of function and fibrosis, and the use of folds of increase can help with external validation. However, there were differences between groups regarding the use of angiotensin-converting enzyme and angiotensin receptor blocker. Despite being a confounding factor, this limitation can be explained by the fact that Group 3 patients had lower bi-ventricular ejection fraction values and, with the presence of a fix afterload generated by severe AS, they had lower tolerance to use of vasodilator drugs. Besides, although there is no evidence of prognostic improvement with guideline directed medical therapy in patients with severe AS and ventricular dysfunction, all patients were on these drugs, at maximum tolerated doses, while awaiting interventional treatment. Another limitation is that BNP and hsTnI are not specific to AS. Also, atrial fibrillation may jeopardize T1-mapping measurements. Nevertheless, incidence of atrial fibrillation was similar between the group and we carefully repeated and averaged T1-mapping measurements, in addition to performing adequate heart rate control, in order to decrease the deleterious effects of atrial fibrillation on these data (16). Although this was a prospective cohort with multimodality evaluation, future studies with a larger number of patients are still warranted to further evaluate the impact of biomarkers in cardiac remodeling in LFLG-AS patients.

## Conclusions

In LFLG-AS patients, group classification according to BNP and hsTnI levels was associated with progressive worsening of imaging parameters of bi-ventricular remodeling and LV fibrosis by CMR, and worse echocardiographic LVEF despite reduction on valvuloarterial impedance. Besides, higher levels of BNP and hsTnI were not associated with flow reserve parameters.

## Data Availability

The original contributions presented in the study are included in the article/**Supplementary Material**, further inquiries can be directed to the corresponding author.
