# Supplementary material for: Multimodality imaging methods and systemic biomarkers in classical low-flow low-gradient aortic stenosis: Key findings for risk stratification
Source: Front Cardiovasc Med. 2023 Apr 27;10:1149613. doi: 10.3389/fcvm.2023.1149613 (PMC10174252; doi:10.3389/fcvm.2023.1149613)
Supplement: Supplementary file 1 [file Table1.docx]

**Supplemental Material**

**Supplemental Table 1. Baseline Clinical and Laboratory Data of the Study Population According BNP Tertiles**

|  | Low BNP  (n=16) | Intermediate BNP  (n=17) | High BNP  (n=16) | P value* |
| --- | --- | --- | --- | --- |
| Clinical Data |  |  |  |  |
| Age, years | 66.9 ± 7.0 | 66.5 ± 9.6 | 69.1 ± 8.9 | 0.66 |
| Body surface area, m² | 1.81 ± 0.15 | 1.80 ± 0.17 | 1.81 ± 0.14 | 1.00 |
| Female gender | 6 (37.5) | 3 (17.6) | 2 (12.5) | 0.21 |
| Diabetes Mellitus | 8 (50) | 5 (29.4) | 5 (31.3) | 0.40 |
| Hypertension | 13 (81.3) | 13 (76.5) | 8 (50) | 0.12 |
| Atrial fibrillation | 4 (25) | 5 (29.4) | 3 (18.8) | 0.77 |
| Angina | 5 (31.3) | 4 (23.5) | 3 (18.8) | 0.71 |
| Previous CABG | 3 (18.8) | 1 (5.9) | 3 (18.8) | 0.43 |
| EuroSCORE II, % | 2.95 ± 2.41 | 2.71 ± 1.77 | 4.63 ± 3.40 | 0.11 |
| STS, % | 3.08 ± 1.93 | 2.89 ± 2.10 | 3.81 ± 2.27 | 0.47 |
| Medications |  |  |  |  |
| ACE inhibitor or ARB | 14 (87.5) | 13 (76.5) | 12 (50) | 0.05 |
| Beta blockers | 9 (56.3) | 7 (41.2) | 10 (62.5) | 0.45 |
| Antiplatelets | 12 (75) | 9 (52.9) | 10 (62.5) | 0.42 |
| Diuretics | 14 (87.5) | 14 (82.4) | 15 (93.8) | 0.60 |
| Statins | 15 (93.8) | 10 (58.8) | 11 (68.8) | 0.04 |
| Digital | 3 (18.8) | 4 (23.5) | 2 (12.5) | 0.71 |
| Oral anticoagulation | 4 (25) | 5 (29.4) | 3 (18.8) | 0.77 |
| Electrocardiogram |  |  |  |  |
| Left Bundle Branch Block | 5 (31.3) | 5 (29.4) | 3 (18.8) | 0.68 |
| Right Bundle Branch Block | 0 (0) | 1 (5.9) | 2 (12.5) | 0.23 |
| Laboratory data |  |  |  |  |
| Hematocrit, % | 41.4 ± 1.1 | 40.6 ± 3.3 | 41.2 ± 1.2 | 0.89 |
| C reactive protein, pg/ml | 3.31 ± 3.44 | 9.26 ± 16.58 | 13.62 ± 18.63 | 0.14 |
| CKD (eGFR <60 mL/min) | 6 (37.5) | 3 (17.6) | 9 (56.3) | 0.07 |
| High-sensitivity troponin I, ng/ml | 0.05 ± 0.02 | 0.12 ± 0.04 | 0.15 ± 0.08 | 0.26 |
| B-type natriuretic peptide, pg/ml | 107.5 (4-177) | 408.5 (191-610) | 1319 (645-2741) | <0.01†‡§ |

Values are mean±standard deviation, median (interquartile range), or n (%). ACE indicates angiotensin-converting enzyme; ARB, angiotensin receptor blocker; CABG, coronary artery bypass graft; CKD, chronic kidney disease; eGFR, estimated glomerular filtration rate.

*Overall *P* value among groups: group 1, group 2 and group 3.

†Significant difference (*P*<0.05) between group 1 vs group 2.

‡Significant difference (*P*<0.05) between group 1 vs group 3.

§Significant difference (*P*<0.05) between group 2 vs group 3.

**Supplemental Table 2. Baseline Clinical and Laboratory Data of the Study Population According to Troponin I Tertiles**

|  | Low  Troponin I  (n=16) | Intermediate Troponin I  (n=17) | High  Troponin I  (n=16) | P value* |
| --- | --- | --- | --- | --- |
| Clinical Data |  |  |  |  |
| Age, years | 67.19 ± 7.83 | 69.41 ± 10.01 | 65.69 ± 7.28 | 0.45 |
| Body surface area, m² | 1.82 ± 0.17 | 1.84 ± 0.13 | 1.76 ± 0.16 | 0.29 |
| Female gender | 5 (31.3) | 2 (11.8) | 4 (25) | 0.37 |
| Diabetes Mellitus | 7 (43.8) | 6 (35.3) | 5 (31.3) | 0.76 |
| Hypertension | 13 (81.3) | 10 (58.8) | 11 (68.8) | 0.37 |
| Atrial fibrillation | 3 (18.8) | 6 (35.3) | 3 (18.8) | 0.45 |
| Angina | 2 (12.5) | 2 (11.8) | 8 (50) | **0.02** |
| Previous CABG | 2 (12.5) | 3 (17.6) | 2 (12.5) | 0.89 |
| EuroSCORE II, % | 2.4 ± 1.7 | 4.1 ± 3.6 | 3.8 ± 2.3 | 0.17 |
| STS, % | 2.7 ± 1.8 | 3.5 ± 2.5 | 3.6 ± 1.9 | 0.41 |
| Medications |  |  |  |  |
| ACE inhibitor or ARB | 15 (93.8) | 10 (58.8) | 10 (62.5) | 0.03 |
| Beta blockers | 13 (81.3) | 6 (35.3) | 7 (43.8) | 0.02 |
| Antiplatelets | 11 (68.8) | 12 (70.6) | 8 (50) | 0.40 |
| Diuretics | 13 (81.3) | 16 (94.1) | 14 (87.5) | 0.52 |
| Statins | 14 (87.5) | 11 (64.7) | 11 (68.8) | 0.26 |
| Digital | 2 (12.5) | 3 (17.6) | 4 (25) | 0.66 |
| Oral anticoagulation | 3 (18.8) | 5 (29.4) | 4 (25) | 0.77 |
| Electrocardiogram |  |  |  |  |
| Left Bundle Branch Block | 6 (37.5) | 1 (5.9) | 6 (37.5) | 0.03 |
| Right Bundle Branch Block | 1 (6.3) | 2 (11.8) | 0 (0) | 0.25 |
| Laboratory data |  |  |  |  |
| Hematocrit, % | 39.6 ± 10.7 | 42.0 ± 5.5 | 41.7 ± 5.3 | 0.77 |
| C reactive protein, pg/ml | 6.4 ± 11.3 | 9.8 ± 14.0 | 9.8 ± 18.9 | 0.77 |
| CKD (eGFR <60 mL/min) | 2 (12.5) | 8 (47.1) | 8 (50) | 0.05 |
| High-sensitivity troponin I, ng/ml | 0.015 (0.01-0.03) | 0.04 (0.03-0.08) | 0.17 (0.08-0.51) | <0.01†‡§ |
| B-type natriuretic peptide, pg/ml | 160 (4-854) | 541 (66-2741) | 580 (100-3583) | 0.03†‡ |

Values are mean±standard deviation, median (interquartile range), or n (%). ACE indicates angiotensin-converting enzyme; ARB, angiotensin receptor blocker; CABG, coronary artery bypass graft; CKD, chronic kidney disease; eGFR, estimated glomerular filtration rate.

*Overall *P* value among groups: group 1, group 2 and group 3.

†Significant difference (*P*<0.05) between group 1 vs group 2.

‡Significant difference (*P*<0.05) between group 1 vs group 3.

§Significant difference (*P*<0.05) between group 2 vs group 3.

**Supplemental Table 3. Baseline Echocardiography and Dobutamine Stress Echocardiography Data According BNP Tertiles**

|  | **Low BNP**  **(n=16)** | **Intermediate BNP**  **(n=17)** | **High BNP**  **(n=16)** | | **P Value*** |
| --- | --- | --- | --- | --- | --- |
| **Baseline Echocardiography** |  |  | |  |  |
| Aortic root, mm | 32 ± 3.4 | 32.8 ± 5.3 | | 33.1 ± 5.5 | 0.81 |
| Left atrium, mm | 41 (38-57) | 44.5 (42-47) | | 49.0 (39-57) | 0.04‡ |
| Interventricular septum, mm | 11.3 ± 2.1 | 11.2 ± 2.3 | | 10.9 ± 2.4 | 0.84 |
| Posterior wall, mm | 10.8 ± 1.4 | 10.5 ± 1.7 | | 9.9 ± 1.9 | 0.32 |
| LVEDV, mm | 57.8 ± 6.3 | 58.1 ± 6.4 | | 60.2 ± 8.1 | 0.58 |
| LVESV, mm | 45.7 ± 5.5 | 45.9 ± 7.6 | | 49.2 ± 9.5 | 0.37 |
| LVEF, % | 38 (26-35) | 35 (20-47) | | 28 (19-45) | 0.02‡ |
| LV mass, g | 146.6 ± 27.2 | 153.9 ± 51.7 | | 154.3 ± 57.5 | 0.87 |
| Stroke volume index, ml/m² | 36.0 ± 10.3 | 36.0 ± 9.4 | | 35.4 ± 8.1 | 0.98 |
| Aortic valve area, cm² | 0.88 ± 0.2 | 0.78 ± 0.2 | | 0.85 ± 0.2 | 0.23 |
| Aortic valve area index, cm²/m | 0.49 ± 0.1 | 0.43 ± 0.1 | | 0.46 ± 0.1 | 0.22 |
| Peak transaortic gradient, mmHg | 42.4 ± 14.5 | 43.9 ± 11.4 | | 43.4 ± 13.6 | 0.95 |
| Mean transaortic gradient, mmHg | 24.9 ± 8.5 | 26.2 ± 7.9 | | 25.0 ± 7.8 | 0.88 |
| Moderate/severe functional mitral regurgitation | 5 (31.3) | 6 (35.3) | | 6 (37.5) | 0.72 |
| Moderate/severe functional tricuspid regurgitation, | 2 (12.5) | 3 (17.6) | | 2 (12.5) | 0.89 |
| Systolic pulmonary artery pressure, mmHg | 42 ± 10.7 | 43.4 ± 13.5 | | 45.4 ± 10.0 | 0.78 |
| Valvuloarterial impedance, mmHg/ml/m² | 5.4 ± 0.8 | 5.4 ± 1.0 | | 4.8 ± 0.9 | 0.15 |
| Global longitudinal strain, [−]% | 10.6 ± 3.1 | 10.2 ± 2.5 | | 8.7 ± 2.2 | 0.14 |
| **Dobutamine Stress Echocardiography** |  |  | |  |  |
| Basal aortic valve area, cm² | 0.88 ± 0.2 | 0.86 ± 0.2 | | 0.81 ± 0.2 | 0.60 |
| Peak stress aortic valve area, cm² | 0.98 ± 0.2 | 0.85 ± 0.3 | | 0.88 ± 0.2 | 0.32 |
| Basal mean transaortic gradient, mmHg | 27 ± 9.9 | 26.3 ± 8.9 | | 26.2 ± 6.8 | 0.96 |
| Peak stress mean gradient, mmHg | 38.4 ± 10.2 | 40.2 ± 18.1 | | 42.8 ± 19.1 | 0.85 |
| Basal stroke volume index, ml/m² | 35.2 ± 8.6 | 29.4 ± 7.2 | | 35.7 ± 22.0 | 0.58 |
| Peak stress stroke volume index, ml/m² | 39.6 ± 9.4 | 43.5 ± 11.2 | | 35.8 ± 8.4 | 0.29 |
| Presence of flow reserve | 12 (75) | 14 (82.4) | | 9 (56.3) | 0.24 |

Values are mean±standard deviation, median (interquartile range), or n (%). LV means left ventricular; LVEDV, left ventricular end-diastolic volume; LVEF, left ventricular ejection fraction; and LVESV, left ventricular end-systolic volume.

*Overall P value among groups: group 1, group 2 and group 3.

†Significant difference (P<0.05) between group 1 vs group 2.

‡Significant difference (P<0.05) between group 1 vs group 3.

§Significant difference (P<0.05) between group 2 vs group 3.

**Supplemental Table 4. Baseline Echocardiography and Dobutamine Stress Echocardiography Data According Troponin I Tertiles**

|  | Low  Troponin I  (n=16) | Intermediate Troponin I  (n=17) | High  Troponin I  (n=16) | | **P Value*** |
| --- | --- | --- | --- | --- | --- |
| **Baseline Echocardiography** |  |  | |  |  |
| Aortic root, mm | 31.8 ± 5.2 | 33.3 ± 5.2 | | 32.9 ± 3.9 | 0.68 |
| Left atrium, mm | 44.9 ± 5.4 | 49.5 ± 5.5 | | 46.9 ± 7.9 | 0.12 |
| Interventricular septum, mm | 10.6 ± 2.1 | 11.8 ± 2.3 | | 11.1 ± 2.0 | 0.29 |
| Posterior wall, mm | 10.1 ± 1.8 | 10.9 ± 1.9 | | 10.1 ± 1.3 | 0.25 |
| LVEDV, mm | 57.6 ± 5.8 | 58.2 ± 7.0 | | 60.3 ± 8.6 | 0.51 |
| LVESV, mm | 45.6 ± 5.6 | 45.7 ± 8.7 | | 49.4 ± 8.2 | 0.56 |
| LVEF, % | 36 ± 6.9 | 33.4 ± 8.6 | | 32.7 ± 8.6 | 0.28 |
| LV mass, g | 132.8 ± 23.6 | 160.5 ± 52.2 | | 161.1 ± 53.9 | 0.14 |
| Stroke volume index, ml/m² | 36.7 ± 9.5 | 37.2 ± 6.4 | | 33.2 ± 11.2 | 0.43 |
| Aortic valve area, cm² | 0.87 ± 0.2 | 0.86 ± 0.2 | | 0.78 ± 0.1 | 0.35 |
| Aortic valve area index, cm²/m | 0.48 ± 0.1 | 0.46 ± 0.1 | | 0.44 ± 0.1 | 0.53 |
| Peak transaortic gradient, mmHg | 41.8 ± 13.8 | 43.0 ± 13.9 | | 45.1 ± 11.5 | 0.78 |
| Mean transaortic gradient, mmHg | 24.1 ± 2.2 | 26.3 ± 2.1 | | 25.7 ± 1.6 | 0.72 |
| Moderate/severe functional mitral regurgitation | 6 (37.5) | 4 (23.5) | | 7 (43.8) | 0.46 |
| Moderate/severe functional tricuspid regurgitation, | 3 (18.8) | 0 (0) | | 4 (25) | 0.03 |
| Systolic pulmonary artery pressure, mmHg | 40.9 ± 12.3 | 40.8 ± 8.6 | | 48.3 ± 10.7 | 0.19 |
| Valvuloarterial impedance, mmHg/ml/m² | 5.15 (4.2-6.2) | 5.00 (2.2-6.4) | | 5.3 (4.4-7.8) | 0.05 |
| Global longitudinal strain, [−]% | 10.0 ± 1.9 | 10.2 ± 2.7 | | 9.4 ± 3.5 | 0.71 |
| **Dobutamine Stress Echocardiography** |  |  | |  |  |
| Basal aortic valve area, cm² | 0.88 ± 0.2 | 0.85 ± 0.2 | | 0.83 ± 0.2 | 0.76 |
| Peak stress aortic valve area, cm² | 1.00 ± 0.3 | 0.88 ± 0.2 | | 0.84 ± 0.2 | 0.27 |
| Basal mean transaortic gradient, mmHg | 25.9 ± 8.3 | 27.2 ± 7.2 | | 26.3 ± 10.2 | 0.91 |
| Peak stress mean gradient, mmHg | 37.4 ± 9.8 | 41.9 ± 15.0 | | 40.5 ± 19.5 | 0.88 |
| Basal stroke volume index, ml/m² | 33.4 ± 7.8 | 31.0 ± 10.8 | | 36.5 ± 21.8 | 0.67 |
| Peak stress stroke volume index, ml/m² | 40.1 ± 7.8 | 39.2 ± 8.4 | | 38.5 ± 12.8 | 0.96 |
| Presence of flow reserve | 11 (68.8) | 13 (76.5) | | 11 (68.8) | 0.85 |

Values are mean±standard deviation, median (interquartile range), or n (%). LV means left ventricular; LVEDV, left ventricular end-diastolic volume; LVEF, left ventricular ejection fraction; and LVESV, left ventricular end-systolic volume.

*Overall P value among groups: group 1, group 2 and group 3.

†Significant difference (P<0.05) between group 1 vs group 2.

‡Significant difference (P<0.05) between group 1 vs group 3.

§Significant difference (P<0.05) between group 2 vs group 3.

**Supplemental Table 5. Baseline Cardiac Magnetic Resonance Data According BNP Tertiles**

|  | Low BNP  (n=16) | Intermediate BNP  (n=17) | High BNP  (n=16) | P Value* |
| --- | --- | --- | --- | --- |
| RVEDV index, mL/m² | 58.9 (37-76) | 59.4 (30-111) | 92 (39-204) | 0.04‡ |
| RVESV index, mL/m² | 22.5 (12-34) | 31 (11-79) | 65 (11-177) | <0.01‡ |
| RV ejection fraction, % | 61 (41-75) | 57 (27-68) | 29 (16-71) | <0.01‡ |
| LVEDV index, mL/m² | 102 (65-158) | 131 (70-169) | 125 (84-188) | 0.02‡† |
| LVESV index, mL/m² | 63 (26-106) | 80 (48-131) | 88 (54-162) | <0.01‡ |
| LVEF, % | 40 (26-64) | 30 (18-45) | 28 (12-49) | <0.01‡ |
| Aortic valve area, cm² | 0.80 ± 0.06 | 0.90 ± 0.07 | 0.87 ± 0.12 | 0.54 |
| Positive transmural delayed-enhancement images | 0.36 ± 0.13 | 0.33 ± 0.13 | 0.42 ± 0.15 | 0.61 |
| Positive mesocardial delayed-enhancement images | 0.14 ± 0.10 | 0.33 ± 0.13 | 0.25 ± 0.13 | 0.32 |
| LV mass, g | 177 ± 11 | 204 (16) | 216 (15) | 0.10 |
| Late gadolinium enhancement mass, g | 11.2 ± 3.3 | 9.3 ± 3.2 | 8.9 ± 2.3 | 0.79 |
| ECV including delayed-enhancement images, % | 29.5 ± 1.5 | 30.3 ± 1.2 | 31.5 ± 1.6 | 0.19 |
| ECV without delayed-enhancement images, %, | 29.0 ± 1.4 | 30.1 ± 1.1 | 30.8 ± 1.8 | 0.50 |
| iECV, ml/m² | 28.5 (17-42) | 38.8 (21.6-51.2) | 38.8 (25.0-56.5) | **0.01†‡** |

Values are mean±standard deviation, median (interquartile range), or n (%). ECV indicates extracellular volume; iECV, indexed extracellular volume; LGE, late gadolinium enhancement; LV, left ventricular; LVEDV, left ventricular end-diastolic volume; LVEF, left ventricular ejection fraction; LVESV, left ventricular end-systolic volume; RV, right ventricular; RVEDV, right ventricular end-diastolic volume; and RVESV, right ventricular end-systolic volume.

*Overall P value among groups: group 1, group 2 and group 3.

†Significant difference (P<0.05) between group 1 vs group 2.

‡Significant difference (P<0.05) between group 1 vs group 3.

§Significant difference (P<0.05) between group 2 vs group 3.

**Supplemental Table 6. Baseline Cardiac Magnetic Resonance Data According Troponin I Tertiles**

|  | Low  Troponin I  (n=16) | Intermediate Troponin I  (n=17) | High  Troponin I  (n=16) | P Value * |
| --- | --- | --- | --- | --- |
| RVEDV index, mL/m² | 58.1 ± 16.8 | 71.9 ± 23.5 | 79.1 ± 43.1 | 0.15 |
| RVESV index, mL/m² | 24.5 ± 14.5 | 42.1 ± 23.7 | 49.7 ± 42.0 | 0.06 |
| RV ejection fraction, % | 64 (28-75) | 45 (17-71) | 40 (16-67) | 0.01†‡ |
| LVEDV index, mL/m² | 99 (65-161) | 123 (91-188) | 137 (78-184) | 0.03‡ |
| LVESV index, mL/m² | 61 (26-120) | 80 (54-162) | 92 (39-162) | 0.03‡ |
| LVEF, % | 40 (25-64) | 33 (14-49) | 29 (12-50) | 0.03‡ |
| Aortic valve area, cm² | 0.80 ± 0.2 | 0.96 ± 0.3 | 0.77 ± 0.3 | 0.17 |
| Positive transmural delayed-enhancement images | 0.27 ± 0.46 | 0.35 ± 0.49 | 0.43 ± 0.51 | 0.67 |
| Positive mesocardial delayed-enhancement images | 0.07 ± 0.25 | 0.29 ± 0.47 | 0.36 ± 0.50 | 0.16 |
| LV mass, g | 184.4 ± 54.7 | 199.4 ± 50.2 | 217.2 ± 51.8 | 0.25 |
| Late gadolinium enhancement mass, g | 7.96 ± 2.2 | 11.10 ± 13.3 | 11.82 ± 11.0 | 0.66 |
| ECV including delayed-enhancement images, % | 28.7 ± 5.9 | 31.0 ± 6.4 | 31.9 ± 6.7 | 0.28 |
| ECV without delayed-enhancement images, %, | 28.5 ± 6.0 | 30.1 ± 5.4 | 30.5 ± 3.4 | 0.56 |
| iECV, ml/m² | 27.4 (17-42) | 38.6 (26-57) | 40.4 (25-54) | 0.01‡ |

Values are mean±standard deviation, median (interquartile range), or n (%). ECV indicates extracellular volume; iECV, indexed extracellular volume; LGE, late gadolinium enhancement; LV, left ventricular; LVEDV, left ventricular end-diastolic volume; LVEF, left ventricular ejection fraction; LVESV, left ventricular end-systolic volume; RV, right ventricular; RVEDV, right ventricular end-diastolic volume; and RVESV, right ventricular end-systolic volume.

*Overall P value among groups: group 1, group 2 and group 3.

†Significant difference (P<0.05) between group 1 vs group 2.

‡Significant difference (P<0.05) between group 1 vs group 3.

§Significant difference (P<0.05) between group 2 vs group 3.
